# Prayer and meditation among Danish first time mothers—a questionnaire study

**DOI:** 10.1186/s12884-016-0802-6

**Published:** 2016-01-19

**Authors:** Christina Prinds, Dorte Hvidtjørn, Axel Skytthe, Ole Mogensen, Niels Christian Hvidt

**Affiliations:** Department of Clinical Institute, University of Southern Denmark, J.B. Winsløws Vej 19, DK-5000 Odense, C Denmark; University College South Denmark, Degnevej 16, 6705 Esbjerg Ø, Denmark; Institute of Public Health, University of Southern Denmark, J.B. Winsløws Vej 9, DK-5000 Odense C, Denmark; Department of Gynaecology and Obstetrics, Odense University Hospital, Sdr. Boulevard 29, DK-5000 Odense C, Denmark

**Keywords:** Motherhood, Prayer, Meditation, Meaning-making, Childbirth, Preterm birth, Existential, Religion, Spirituality

## Abstract

**Background:**

Mothers’ existential dimensions in the transition to motherhood have not been described thoroughly. They might experience disruption and new perspectives in existential ways and this may especially be the case in preterm birth. The aim of this study was twofold. First we investigated the existential dimension of motherhood transition in a secularized context, through practices of prayer and meditation. Second we described the relationship between time of birth (term/preterm) and the prayer/meditation practices of the mothers.

**Methods:**

Data were gathered from a nationwide questionnaire survey among first time mothers conducted during the summer 2011. All Danish women who gave birth before the 32^nd^ pregnancy week (*n* = 255), and double the number of mothers who gave birth at full term (*n* = 658) in 2010 were included (total *n* = 913). The questionnaire consisted of 46 overall items categorized in seven sections, which independently cover important aspects of existential meaning-making related to becoming a mother. The respondent rate was 57 % (*n* = 517).

**Results:**

Moments of praying or meditation 6–18 months post partum were reported by 65 %, and mothers who responded affirmatively, practiced prayer (*n* = 286) more than meditation (*n* = 89), *p* < 0,001. We did not observe differences in affirmative responses to prayer or meditation between mothers of full term or preterm born children, not even after controlling for perinatal or post partum loss, mode of birth, age, status of cohabiting or education.

**Conclusions:**

In this explorative study we found specific practices of existential meaning-making through prayer and/or meditation among first time mothers, living in a very secularized context. Yet we know only little about character or importance of these practices among mothers, and hardly anything about existential meaning-making among new fathers. Hence the implications of meaning-making practices related to other dimensions of health are difficult to address in a qualified way in care for new mothers and families.

## Background

### Beginning of life and end of life

In recent decades there has been an increasing amount of research on the manifold forms of making meaning at the end of life among patients and relatives. Some of this research addresses existential, spiritual or religious considerations and needs, and suggests that they intensify when facing the end of life [1, 2]. In spite of this, research suggests that such considerations are not met adequately in the health care system [3, 4].

Spiritual ways of making meaning is a focus in the field of palliative care related to the end of life, but it is not addressed at all in maternity services in Denmark [5, 6]. In relation to the beginning of life, health care research related to existential meaning-making remains limited, even in the paradoxical situations where the beginning and end of life sometimes coincide, for example in some cases when giving birth preterm. Previous research suggests that the impact of considerations related to the beginning of life may be similar to those at the end of life. These events are gateways that encircle life, and both of them may facilitate considerations related to existential meaning making [7, 8]. This study concentrated on a specific practice of existential meaning-making, namely prayer and/or meditation when becoming a mother for the first time in a secular context.

### First time motherhood at term or preterm

Motherhood as an existentially transformative process has been explored in psychological and anthropological research [9–11]. A recent literature review argues that the joy of birth has been neglected in literature, thus leaving out any spiritual or sacred meaning [12]. Childbirth has been understood as a journey into *“…motherland”* [13] *“… a miracle”* [14] *“… a religious act”* [15] and even as *“…a sacrament”* [16]. In a society known to be the most secular in the world [17], it may seem provocative to study first time motherhood in such terms. Recent Danish research among other groups of patients, though, has found reflections related to existential meaning-making to be intensified during illness. In relation to cancer, a recent survey among patients with severe lung cancer showed that 64 % had some belief in God and/or a spiritual power [18]. An interview study among young leukemia patients found that existing beliefs related to meaning-making (both religious and non-religious) prior to cancer were strengthened during illness [19]. Ausker described one of her informants having experienced becoming a mother as a life threshold similar to her experience of leukemia [19, p. 132]. On the basis of the latest European Value Survey (EVS) among 1507 Danes in 2008, Andersen et al. analyzed the religiosity of Danes in a lifetime perspective. They found beliefs to be quite stable, independent of age, but sensitive to intense life events, as for example childbirth or serious illness [20].

Giving birth preterm may be even more existentially transformative since the period of the beginning of life is potentially confronted with the end of life. Mothers of preterm born children may suffer from post-traumatic stress and anxiety [21], and some are found to develop potentially problematic attachment styles in the mother-child relation [22]. Preterm birth is the leading cause of perinatal mortality and morbidity globally [23], which, in many countries ensures special care of parents in maternity services. This is also the case in Denmark [6]. The impact of preterm childbirth on motherhood was studied in Denmark among parents of 66 neonates in 2008. Among the mothers, 30 % met the criteria for Post Traumatic Stress Disorder (PTSD) either clinically or sub-clinically [24]. In relation to motherhood of preterm born children in general, practices of existential meaning-making have not previously been studied in Danish health care research. Other aspects of preterm childbirth in Denmark have been studied recently, for example the challenge concerning the construction of motherhood related to a moderate preterm child [25]. Also the phenomenon of christening of newborns in acute situations at the hospital, has been explored and found to be potential significant to parents [26].

### Prayer and meditation practice

Existential meaning is a multidimensional construct explored in different ways. Whereas religion in an earlier European context was an obvious and normal socio-cultural source of meaning during illness, it seems to have lost its importance in western medicine today [27]. The increased focus on science-based and evidence-based medicine might also induce a more private process of making meaning, since issues of an existential character are not obviously important in medical research [28, 29]. To study existential meaning-making, Danish researchers, on the basis of a literature review, have proposed a new conceptual framework for existential meaning-making, consisting of three dimensions: cognition, practice and importance [30]. In this study we focus on the practice dimension of existential meaning making, especially religious or spiritual practices, namely prayer and meditation.

Meditation practices have been studied in a randomized controlled trial among nulliparous women in Denmark, focusing on the effect of the practice of hypnosis during birth in relation to pain and the experience of childbirth. The study showed that overall, women randomized to hypnosis had a better childbirth experience than the two control groups [31]. In antenatal education classes in Denmark, there has been a tradition of spiritually oriented practices like visualization and mindfulness and yoga [32, 33].

Prayer and/or meditation practices have been studied among other groups in Denmark. 480 hospitalized people and their relatives at Copenhagen University Hospital (Rigshospitalet) participated in a questionnaire survey about faith in 2006. Sixty-two percent replied affirmatively to the question: “Do you take some moments of prayer, meditation or contemplation or something like that?” [34] When asked in population based values surveys, Danes do not pray much. According to the EVS in 2008, 50 % percent responded to the statement “How often do you pray to God outside of religious services?” with 9 options ranking from *Every day* to *Never*, the choices being dichotomized between *Less often* and *Never*. Of these answers, 55 % were in the *“A few times a year”* or *“Less often than a few times a year”* options. 48 % reported that they never prayed [35].^1^ When asked “Do you take some moments of prayer or meditation or something like that?” 48 % replied affirmatively and 51 % replied negatively [35]. In a Danish questionnaire survey exploring familial resemblance in religiosity among 6707 participants of comparable age from 2009, 48 % gave a positive answer to the statement “How often do you pray to God outside of religious services?” 51 % reported that they never prayed. The mean age in the study was 29 years, 60 % of the respondents being women [36]. There seems to be a difference between responses from hospitalized patients and relatives and the more population-based surveys on prayer, indicating that a practice such as prayer or meditation increases when being hospitalized.

Mothers in other studies, based on data from primarily Western countries, giving birth is highlighted as something holy, a spiritual experience or blessed event [15, 16, 37]. We have no knowledge as to whether Danish mothers turn to religious or spiritual dimensions of making meaning of life, including practices of prayer and meditation, or whether there are different practices between mothers giving birth at term or preterm. Hence we do not know if or how to address this dimension of life and health in the maternity service, or whether we should differentiate between care for mothers of preterm born children or children born at term. The aim of this study was thus firstly to investigate practices of prayer and meditation among Danish first time mothers, and secondly to distinguish between mothers who gave birth at full term (FT) or preterm (PT) in terms of their respective prayer/meditation practices. On the basis of previous research related to preterm birth, we hypothesized mothers of preterm born children would practice prayer/meditation more than mothers of full term born children. On the basis on existing research related to prayer and meditation, we hypothesized that meditation practices would be more widespread than prayer among Danish first time mothers.

## Methods

We used data from a cross sectional questionnaire survey among Danish first time mothers who gave birth in 2010. We retrieved information from the Danish Medical Birth Register about all mothers who gave birth before the 32nd week of gestation in Denmark and twice that number of women who gave birth at full term, randomly sampled. Mothers experiencing perinatal or post partum loss (stillbirth after the 22^nd^ pregnancy week or death post partum, covering up till 100 days post partum) were also included. Mothers of twins, who lost one of them, were categorized as mothers of a living child. In the present paper, mothers who gave birth at full term will be called *full term (FT) mothers),* whereas mothers who gave birth before the 32^nd^ pregnancy week go as *preterm (PT) mothers*. We included only first time mothers who had given birth in 2010, in order to reduce the risk of recall bias if the experience of becoming a mother was older than 18 months. The free of charge antenatal care in Denmark encompasses almost all pregnant women (99 %) [38], which makes the national registration almost complete.

In July 2011, a postal questionnaire about existential meaning making in motherhood was sent to all participants along with a covering letter. Reminder letters were sent 10 weeks later [39]. No incentives were given. The Regional Research Ethics Committee of South Denmark approved the study, and the exact registration number from The Danish Data Protection Agency was submitted in the covering letter, which also explained the voluntary nature of participation and the right to withdraw at any time [40]. In line with national regulations participation was obtainment of informed consent [41]. Responses were scanned by a consultancy company (UNI-C), and delivered to researchers in STATA-format and, for long accounts, in SPSS-format. We conducted random checks for consistency between scans and the original questionnaires. All questions from validated questionnaires are reported in an original translated version, and translation of the remaining questions into English were done firstly by the first author, and secondly by a professional translator (MC).

### Survey construction

The questionnaire consisted of 46 general items categorized in seven sections, which independently cover the most important aspects of existential meaning-making related to becoming a mother and the pregnancy, birth and post partum periods respectively. The questionnaire was structured to differentiate between different forms of meaning-making. Existential meaning-making is an overall concept linked to both secular, religious and spiritual orientations. Especially in Northern Europe it is also derived from more secular viewpoints [30]. As explained in the introduction, in 2010 the Danish researchers, la Cour and Hvidt, proposed a conceptualization in which the *existential* is the broader concept, encompassing both existential philosophical values and ideas without any transcendent foundation (secular), as well as meaning orientations related to spiritual and/or religious notions [30]. They propose to apply the three domains: *knowing, doing and being* to the three dimensions of secular, spiritual and religious meaning-making within a “matrix”, to explore consistency between the concepts of cognition, practice and importance. The three concepts of *knowing (cognition), doing (practice) and being (importance)* were introduced by the sociologist Fishman [42] and have been found to have good explanatory power in the analysis of the Danish items related to meaning-making in the European Values Study [43].

The structure of the questionnaire was based on this “matrix”, as well as an underlying understanding of the pregnancy, birth and post partum periods as periods that may actualize existential reflections either partially or as a whole. In the interpretation of findings we also relied on this underlying understanding. As earlier mentioned, we focused in this particular study on those items in the questionnaire that concerned the practice dimension, in particular prayer and meditation.

The items in the questionnaire were developed on the basis of a pilot study, a qualitative interview study among Danish mothers of preterm born children in 2008 [44], as well as on the basis of various other surveys, for example items from the EVS (2008), the Views and Values Survey from the Danish Twin Registry (2009) and the third edition (2008) of the survey concerning religion from the International Social Survey Programme [45]. Items were identified also from an extensive literature review focusing on the existential dimensions of the transition to motherhood among new mothers in other Western oriented countries [46]. According to Nørregaard-Nielsen and Østergaard [47], draft questionnaires were tested among midwifery and public health researchers. On the basis of this process, the questionnaire was revised and then pilot-tested through face-validation with new mothers in one focus group interview and six individual interviews [40, 48].

### Core question

In this paper we focus on and differentiate the item “Do you take some moments of prayer, meditation or contemplation or something like that?” (See item 28 below). This item was designed to provide knowledge about religious and spiritual practices among Danish mothers. It has been used in the EVS 2008 and in a survey among hospitalized patients and their relatives at the Danish Rigshospitalet [34]. In contrast to the previous use of the item, in this questionnaire the options for answering the question were specified, partly to distinguish between religious and spiritual practices and partly to explore how Danish mothers understand such practices. The respondents were allowed to select one or more of the different forms of praying or meditation. Furthermore, we looked into three supplemental items (18, 22 & 25), which were used previously in the EVS or ISSP to search for internal consistency between the dimensions of knowing, doing, and being. Consistency between the dimensions is represented when for example respondents who accept prayer as a dialogue with God (item 28), are also admitting to some kind of belief in a God (item 25) (See Table 1).

**Table 1:** Core question

Item	Question
28	Do you take some moments of prayer, meditation or contemplation or something like that? (no/yes)
	If yes, please explain using the phrases below how you understand prayer or meditation:
	Prayer in church or at other religious gatherings
	Prayer as an inner dialogue addressed to God
	Prayer as an inner dialogue addressed to ‘something greater than myself’
	Prayer as a physical act, e.g. kneeling, folding your hands
	Prayer as music, e.g. hymns, spirituals
	Meditation as a physical activity
	Meditation as a spiritual activity
	Meditation as a means to maintaining or achieving good mental health
	Something else, please state what:
	Three items concerning existential reflections in the knowing- and being dimensions:
18 (being)	Independently of whether you go to church or not, would you say you are…
	A religious person
	A non-religious person
	A convinced atheist
	Don’t know
22 (doing)	How often do you pray to God outside of religious services? Would you say…
	Every day
	More than once a week
	Once a week
	At least once a month
	Several times a year
	Less often
	Never
	Don’t know
25 (knowing)	Which of these statements are closest to your own idea of God?
	I don't believe in God
	I don't know whether there is a God and I don't believe there is any way to find out
	I don't believe in a personal God, but I do believe in a Higher Power of some kind
	I find myself believing in God some of the time, but not at others
	While I have doubts, I feel that I do believe in God
	I know God really exists, and I have no doubts about that
	I don't know

**Table 2:** Characteristics of respondents versus non-respondents, numbers (column percentages) of Danish first-time mothers invited to a cross-sectional survey on existential meaning-making in 2011

	Non-respondents Respondents *n* (%)	Respondents *n* (%)	*P*
	396 (43)	517 (57)	
Term mothers	276 (70)	382 (74)	0.16
Preterm mothers	120 (30)	135 (26)	
Age, years mean	396 (27.9)	517 (29.6)	<0,001
Married/civil partnership	174 (44)	217 (42)	0.52
Not married, cohabiting^a^	214 (54)	294 (56)	
Membership of the Danish			
national church: Yes	283 (72)	423 (82)	<0.001
No	113 (28)	94 (18)	
Caesarean birth	119 (30)	145 (28)	0.88
Vaginal birth	277 (70)	372 (72)	
Mothers not experiencing perinatal or post partum loss	358 (90)	482 (93)	0.12
Mothers experiencing perinatal or post partum loss	38 (10)	35 (7)	

^a^Widows and divorced mothers not included (*n* = 14)

### Statistics

Data was analyzed using Stata version 13.0 [49]. Categorical data were analyzed using the chi-square test of independence and binomial regression was used to control for confounding for perinatal or post partum loss and mode of delivery. Difference in age was tested using a two-sample *t*-test. *P* < 0.05 was considered statistically significant. Respondents answering, *“don’t know”* were included in all analyses, whereas missing or faulty answers were omitted in analyses [50].

We reduced the item concerning prayer to God outside religious services (item 22) into four subgroups. Firstly, one group praying regularly, divided between *“at least once a month”* and *“several times a year”*. Secondly, the biggest group of respondents, reporting prayer several times a year or less often than that, and thirdly, a group reporting *“never”*. We reduced the item concerning the idea of God (item 25) into four subgroups as well. The first three subgroups based on each of the first three possibilities of answers and the fourth subgroup based on a sum of the last three possibilities: first, one group of non-believers; second, a group of agnostics; third, (the biggest group) reporting belief in a higher power; and fourth, a group reporting belief in some kind of god.

## Results

**Fig. 1:**
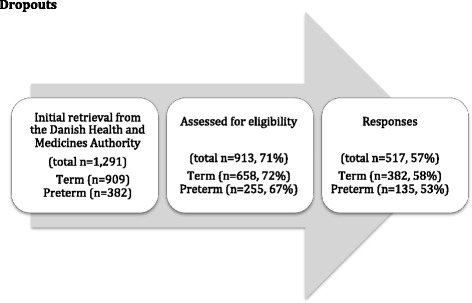
Dropouts

The initial retrieval of 1291 mothers was reduced by 378, either because of a research or address protection (*n* = 370), or caused by emigration or termination of pregnancy before the 10th week (*n* = 8). Research or address protection can be obtained easily in Denmark due to the Danish Act on Processing of Personal Data. Of the 913 mothers included, 517 responded, preterm mothers accounting for 26 % and full term mothers accounting for 74 %. See the dropout flow chart (see Fig. 1).

In relation to differences between respondents and non-respondents, no statistically significant difference was found related to marital/cohabitant status or mode of birth, defined by caesarean birth versus vaginal birth (*p* = 0.88) or neonatal outcome (*p* = 0.12). Also there was no statistically significant difference related to time of birth (full term = 58 % vs. preterm = 53 %) (See Table 2). Eighty-two percent of the respondents were members of the Danish national Church compared to 72 % of the non-respondents with a statistical significant difference between respondents and non-respondents (*p* < 0.001).

### Respondents

A total of 517 participants with a mean age of 29.6 years returned a completed questionnaire. We did not find statistically significant differences between full-term FT and PT mothers in relation to age, educational level or way of living. Eighty-nine percent reported living with a partner. Fifty-seven percent had completed a bachelor’s or master’s degree, whereas 9 % reported having no vocational education at all. However, we did find differences in the mode of birth and the experience of perinatal or post partum death, since the prevalence of caesarean births and loss of a child are higher among PT mothers. Overall, 28 % had a caesarean section, and 7 % experienced the loss of the child with statistically significant differences between the two groups (see Table 3).

**Table 3:** Characteristics of full-term mothers versus preterm mothers, numbers and column percentages

	All *N* (%) (*n* = 517)	FT mothers *N* (%) (*n* = 382)	PT mothers *N* (%) (*n* = 135)	*P*
Socioeconomic factors				
Age, years mean	29.6	29.5	29.9	0.44
Living with a partner	461 (89)	341 (89)	120 (88)	0.72
Bachelor level	157 (32)	113 (31)	44 (35)	0.88
No vocational	47 (9)	36 (10)	11 (9)	
education Short	167 (34)	125 (34)	42 (33)	
Bachelor level	157 (32)	113 (31)	44 (35)	
Master level	122 (25)	92 (25)	30 (23)	
Obstetric factors				
Caesarean section	145 (28)	80 (21)	65 (48)	<0.001
Having experienced perinatal or post partum loss	35 (7)	1(0.3)	34 (25)	<0.001
Spiritual basic factors				
Religious person	287 (56)	212 (56)	75 (56)	0.86
Prayer to God outside service, at least once a month	105 (21)	79 (22)	26 (20)	0.7
Prayer to God outside service, approx. once a year	195 (39)	140 (38)	55 (42)	0.7
Idea of God, believing in higher power	181 (37)	137 (38)	44 (34)	0.43
Idea of God, believing in God	162 (33)	114 (31)	48 (38)	0.43

More than half (56 %) of the mothers reported being a religious person (in the Danish version the wording is more similar to *“a believing person”*), and 33 % reported some kind of belief in God, when asked what their own idea of God was. Belief in some kind of higher power was reported by 37 %. Praying outside religious services once a month or more was reported by 21 % and 39 % reported prayer as rarely as less often than once a year. There were no statistical significant differences between FT and PT mothers in the three items concerning beliefs.

### Prayer and meditation

Of the 517 respondents, 334 (65 %) confirmed having prayed or meditated, with a difference between FT and PT mothers (*p* = 0.05) which vanished when controlling for perinatal or post partum loss and mode of birth (*p* = 0.4). RR of prayer/meditation related to perinatal or post partum loss alone was 1.25 (data not shown). Of the mothers, 166 denied having prayed or meditated and 17 did not answer the question. The mothers practiced prayer rather than meditation (286 versus 89 mothers, *p* < 0,001) See Tables 4 and 5.

**Table 4:** Core question: Do you take some moments of prayer, meditation or contemplation or something like that? Relative risk proportion (RR) of PT mothers 96 (71 %) versus FT mothers 238 (62 %)

Item 28 (95 % CI)	RR	Adjusted RR^a^	Adjusted RR^b^
Prayer and/or meditation	1.14 (0.99–1.30)	1.08 (0.92–1.26)	1.06 (0.91–1.25)

^a^Adjusted for perinatal or post partum loss

^b^Adjusted for perinatal or post partum loss and mode of birth

**Table 5:** Prayer or meditation among first time mothers in Denmark, confirming to have prayed and/or meditated

Prayer	Meditation		
	No	Yes	Total
No	11	37	48
Yes	234	52	286
Total	245	89	334

**Fig. 2:**
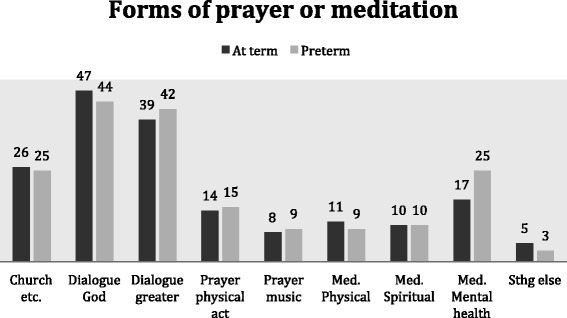
Preferred forms of prayer/meditation among Danish first time mothers. Percentages of the total number of mothers responding affirmatively to prayer/meditation respectively are listed in each bar. See full title of each bar in Table 1

When dichotomizing the options of prayer or meditation, we found no statistically significant differences between FT mothers and PT mothers in their choices of prayer (*p* = 0.86) or meditation (*p* = 0.30) (data not shown). Respondents could choose several of the different forms of prayer or meditation. 11 did not choose any of the options, even though they had said that they prayed or meditated in the introductory question. Among the different forms of prayer, most mothers responded to the option of *“prayer as an inner dialogue addressed to God”* (46 %), followed by *“prayer as an inner dialogue addressed to something greater than myself”* (40 %). In relation to meditation, most mothers responded to the option *“meditation as a means to maintaining or achieving good mental health”* (19 %). We conducted sub-analyses by investigating every possibility of praying or meditation, and found no statistically significant differences in responses from FT and PT mothers in any the items. See Fig. 2.

## Discussion

In the discussion, we address firstly the number of mothers responding affirmatively to prayer/meditation. Secondly, we address the differences when separating prayer and meditation in their answers, and thirdly, the lack of differences between FT and PT mothers.

In this cross-sectional survey among first time mothers in a secular society, we found that 65 % of the mothers responded affirmatively when asked if they had prayed or meditated. This is higher than the 2008 EVS, where 48 % women (pregnancy status unknown) responded affirmatively to the same question [35]. It is also higher than the survey among patients and relatives at Rigshospitalet, where 62 % participants responded affirmatively to the question [34]. In relation to the analyses of data from the EVS, it is suggested that having a child may be one of those life events that intensify religiosity [20]. This is found in a US longitudinal study focusing on lifelong religious development as well [51]. Andersen and colleagues find the doing-dimension to be stable (measured by church attendance), whereas the dimension of “knowing”, for example the conviction that the church is where their spiritual needs are best met, is affected by significant life events [20]. The first time mothers in this survey are not hospitalized, but seem to be engaged in existential meaning-making in a very concrete way. Prayer and meditation are concrete “doing” practices related to a transcendent dimension. The consent to such practices by the majority of the respondents challenges our understanding of these practices being mainly present in relation to traumatic life events such as sickness or death, including traumatic birth.

The respondents in this survey are mothers, not fathers, and perhaps mothers are more attuned to prayer and meditation? In 2012, the sociologists Trzebiatowska and Bruce proposed childbirth as one of the possible reasons for women being more religious than men [52]. One aspect is the focus on birth in most religions, which naturally brings women more in contact with religious reflections and rituals, e.g. christenings. Another aspect highlighted by Trzebiatowska and Bruce is the focus on the biological body in relation to pregnancy and childbirth, a focus seen in several religious settings, e.g. in relation to the regulation of food or sexuality. It is a focus likewise discernible in more spiritually oriented environments, as for example yoga or healing, where health-related benefits seem to be an important motivational factor for participating [52, 53]. This last aspect of the focus on the biological body fits the data in this survey, where the main motivational cause of meditation among respondents was the urge to achieve good mental health (19 % reported this). In this perspective, meditation can be seen as a lifestyle activity in line with healthy food and a fit body in order to stay balanced and energetic. Perhaps Danish mothers pray or meditate due to the ostensibly natural connection between birth and religious rituals, but it seems that more women consent to prayer or meditation than actually participate in religious rituals. This points to an intrinsic and individualistic religious and spiritual orientation – prayer or meditation are not connected to a special community, but can also be practiced alone when pregnant or as a way of coping or caring when giving birth or in the postnatal period [54]. An individualistic approach was also found in a Dutch nationwide survey comparing different kinds of praying. Banziger et al. argue likewise that their finding of a high affirmative response to what they describe as “meditative and impulsive prayer”, is characteristic in secularized society, because of the individualized and non-institutionalized forms of religiosity and spirituality [55].

When separating prayer and meditation, this individualism in a prayer- or meditation practice persists. The initial hypothesis of more mothers responding affirmatively to meditation than prayer, due for example to the focus on spiritually oriented practices in antenatal classes, was not supported. The majority of mothers reported having prayed (286 versus 89), and among them, most endorsed the statement of having addressed their prayer to a personal God. However, in the background question on prayer to God outside religious services, only 21 % agreed, which supports the notion that questions loaded with a conventional understanding of religiosity as church attendance lead to more reluctant responses.

Andersen and Lüchau highlight the apparent paradox that religion has become more important to Danes, but individualized as well, when looking at religious data from the European Values Study. It seems that many contemporary Danes combine seeking answers in religion with doing this in their own individual way: there is a growing confidence in the church providing relevant answers to existential questions, but a receding belief in the church as a special authority [56]. In line with this, Danish researchers suggest Danes have trouble finding comfort in traditional religious beliefs, as Rosen found in a qualitative study in 2008 among Danes in the Copenhagen area: *“I’m a believer – but I’ll be damned if I’m religious”* (Rosen 2009:161). When asked about their idea of God (the knowing dimension), more mothers in this survey reported belief in a higher power (37 %) than belief in some kind of God (33 %). “A higher power” is seen as a spiritual dimension of existential meaning-making. This points to some inconsistency according to “the matrix” structure of the questionnaire, since most mothers at the same time affirmed some practice of prayer (the doing dimension), which is interpreted as a religious way of existential meaning-making. When most mothers report praying, this finding clashes to some extent with the expected widespread move from religious to more spiritual practices, including from prayer to meditation, predicted, for example, by two sociologists claiming a “spiritual revolution” on behalf of belief in a personal God [57]. After all, our findings fit in with other recent Danish data suggesting that Danes have not entirely left religious beliefs and practices in favor of practices of a more spiritual nature, of which meditation is an example [58].

One reason for the preference for praying rather than meditating among these new mothers may be that people in life transitions are emotionally dependent on strong relations. On the basis of neuroscientific research, Danish researchers argue, that improvised prayer is comparable to normal interpersonal interaction [59]. In prayer, the conceived *relation* to a God greater and beyond oneself may be a higher good than that gained in secular meditation practices, often characterized by contemplation within oneself [60]. For the new mother, the relation to her child has already had immense impact on her life, and perhaps, therefore, relating not only to a transcendent *dimension* (as in meditation), but also to a transcendent *relation* (as in praying to someone outside oneself) becomes important [61]?

So - at the same time as conventional religious beliefs are decreasing, women in this survey seem to practice praying as an inner dialogue with either a personal God or *“something bigger than myself”*. There seems to be a combination of the highly individualistic way of making existential meaning of life combined with a widespread use of religious and spiritual practice deriving from a conventional religiously affiliated way of making meaning.

We looked into prayer and meditation practices among first time mothers of both preterm and full term children. Research suggests that traumatic childbirth, for example giving birth preterm, may actualize existential reflections, including religious practices, which ought to be addressed clinically [62]. Therefore, we expected to find a statistically significant difference in the use of prayer and meditation practices. We found a difference (not statistically significant though) between FT and PT mothers, however it vanished when controlling for perinatal or post partum loss and mode of birth. It seems that the loss of a child to a significant degree determined the finding related to prayer/meditation among PT mothers. When looking into specific ways of practicing prayer and meditation, we did not find any differences between FT and PT mothers either. The lack of difference contrasts with other findings from studies comparing experiences from FT and PT parents, showing PT parenthood to be traumatizing also in a long-term perspective [63–65]. Some studies find no differences when comparing FT and PT mothers, for example in relation to hopelessness, where ways of coping after having given birth have been compared in a Polish study [66].

In relation to prayer/meditation, the impact of childbirth and becoming a mother seem to affect the mothers in this study to equal extents. Although we did not find any statistically significant differences between FT and PT mothers in the study, it is obviously still possible that they differ in underlying motivations. Prayer and meditation practices may be motivated by a variety of feelings and experiences. Feelings of grief, loss and betrayal are described among mothers of preterm born children [67, 68], and may motivate prayer and meditation practices, as well as the described feelings of gratitude and joy when becoming a mother [12, 14].

### Limitations

Of the 1291 mothers, only 71 % could be contacted, mainly due to address and research protection. The final respondent rate of 57 % may seem low, but is not unusual. The 2009 Views and Values Survey from the Danish Twin Registry had a respondent rate of 55 %, even though twins are usually willing participants [69]. We sought to resist selection bias through the drop-out analysis, thereby making the differences between respondents and non-respondents transparent [48]. It is seen that more respondents than non-respondents are members of the Danish National Church. Researchers propose Danes to be ‘individualized’ Christians where public religiosity and shared beliefs have diminished [70, 71]. This is reflected for example in churchgoing. Generally 78.4 % of the population are members of the national church (2014), but only 10 % attends church regularly (once a month ore more) [70, 72]. In our sample 6.6 % report going to church regularly, which is even less than the average, and interpreted as an expression of membership of the church being more ‘cultural’ motivated than motivated by a public and shared religiosity [70, 73].

The apparently high proportion of respondents replying affirmatively to prayer and meditation, and the lack of differences between preterm and full term mothers, may be due to the time frame, since the mothers in this study gave birth 6–18 months prior to the survey. Answers might have differed if mothers had been asked earlier in the post partum period, assumedly increasing the difference between FT and PT mothers. The use of prayer and/or meditation has been investigated earlier in Denmark among other groups of people, but not the distinction between different kinds of religious activities, which renders a comparison of the data impossible. Due to the cross-sectional design, changes over time in answers cannot be compared either [74]. We still lack knowledge of the frequency of religious or spiritual practices among new mothers, of their motivation to practice prayer/meditation, and of the content or significance of prayer/meditation in relation to making meaning of having become a mother.

## Conclusions

Firstly, we found that 65 % of Danish first time mothers reported having had moments of prayer or meditation 6–18 months post partum. Secondly, when separating prayer and meditation in the core question, we found that mothers practiced prayer more than meditation. Amongst the five prayer options, most of the practicing mothers chose the option, *“prayer as an inner dialogue addressed to God”* (46 %). Amongst the three meditation options, most mothers chose the option, *“meditation as a means to maintaining or achieving good mental health”* (19 %). Thirdly, we found no differences in the frequency of prayer or meditation between full term and preterm mothers when controlling for perinatal or post partum loss or mode of birth.

It seems that specific practices of existential meaning-making, namely prayer and/or meditation, are indeed found among Danish first time mothers, and yet we know only little about the character or importance of these practices. To do so, qualitative research is needed to supplement this explorative overview. Also more research is needed to include attitudes and the nature of existential meaning-making among new fathers.

## Footnotes

1 In the preparation of this article, data from The Danish Views and Values Survey 2008 is used. Originally professor Peter Gundelach, Institute of Sociology, University of Copenhagen, conducted the survey. The data material was available through ‘Danish Data Archive’ (number DDA-21432). Prinds et al. are responsible for the analyses and interpretation of data in this paper.
